# Castration Tumors in Swine

**Published:** 1901-04

**Authors:** H. Duncan

**Affiliations:** West Union, Iowa


### CASTRATION TUMORS IN SWINE.

By H. Duncan, M.D.,
WEST UNION, IOWA.

There is a disease of swine occurring chiefly in the male, following castration, characterized by a peculiar and distinctive fibrous growth which develops in the castration wound.

It is a not uncommon affection among the swine herds in the Southwest, as Kansas, Missouri, and Nebraska. As to the percentage of animals affected it is difficult to say, but it is possible that about 5 per cent. of the male animals are affected. Most of them never come to market. Those that come to the stockyards are condemned by the Government inspectors, who call the affection scirrhous tumors of the cord. A small fraction of those coming to the yards escape the eye of the inspector, as shown by the statistics furnished by Dr. Day, Government inspector at the Viles Robbins' packing-house. Of 143,568 hogs inspected by Dr. Day during the months of July and August, 1898, twenty-three were condemned on account of suppuration swellings in the scrotum. These figures show that it is quite rare among the swine slaughtered in the great packing-houses.

The clinical history of the animals affected is about as follows: Within two or three weeks after castration a swelling appears, occupying the position of the testicle. Usually but one side is affected; occasionally two tumors make their appearance, merging later into one growth or remaining as separate tumors. The tumor, as a rule, grows rapidly, and in from three to five weeks after the inception of the growth one or more ulcers appear, usually in the cicatrix. Close inspection shows the ulcers to be the external openings of fistulous tracts, from which a thick, light-yellow pyoid material exudes in small quantities—sometimes in large amounts. Sometimes there is a serous fluid, constant but in small amount. The ulcers rarely heal. The growth frequently acquires enormous proportions, as compared with the weight of the hog, and in such cases usually drags upon the ground.

During the earlier stages of the disease the animal has a good appetite and thrives. A noticeable action of the animal thus affected is the constant switching of the tail. Later, when on account of the weight and position the tumor becomes an encumbrance, the animal loses flesh and grows very slowly, but continues to have a good appetite. Still later in the disease, when the tumor becomes a positive load to carry or drag about, the hog has a cachectic appearance. The animal usually dies a violent death, being killed by the owner, or run over by a wagon, or trampled by cattle; otherwise it usually dies within twelve to eighteen months from the appearance of the growth. The fact that in the later stages the animal is unable without considerable effort to obtain food and drink may hasten its death. The growths have never been observed to heal spontaneously. Much more rarely a similar growth is observed in the female, usually on the breast.

The growth appears to be invariably in connection with the spermatic cord, and is in most cases single, but there may be two or more masses on the same side of the scrotum. It consists of a softened centre enclosed by a capsule of varying thickness of firm, vascular, white, fibrous tissue. Externally there is no distinct capsule, the firmer fibrous tissue merging more or less gradually into loose fibromyxomatous tissue. The growth, when large enough, invades the surrounding muscles. The skin covering the tumor is firmly adherent and always includes the scar from castration. The form of the swelling is generally round or oval. The larger may present various-sized protuberances and irregularities. Not infrequently there are smaller secondary tumors or nodules in close relation to the primary growth, varying in size from a pea to as large as a hen's egg. These secondary nodules have a structure similar to that of the primary growth. Occasionally several small tumors are found scattered along the course of the spermatic cord. In all cases the interior of the growths contains one or more cavities. The size of these vary greatly, and bear no definite relation to the size of the growth. A tumor weighing five kilos may contain an area of softening as large as a walnut, the rest of the tissue being firm and white, or there may be a huge cavity containing a litre or more of turbid, thick fluid, the fibrous wall being from 4 to 16 cm., more or less, in thickness. Even the smallest nodules, no larger than a pea, contain a central area of softening. The contents of the cavity may be a soft, spongy, yellowish material adherent to the walls, or there may be a thin or thick turbid yellowish fluid; between these extremes are all possible transitional appearances. The necrotic material has a penetrating, somewhat sweetish, rather sickening odor, which is very characteristic. The margins are, as a rule, abrupt and generally the solid tissue shows a more or less brownish discoloration for 0.5 cm. or so away from the margins.

During the summer of 1898 there was an opportunity for removing a number of specimens from the live animal. The following spring a series of tumors were obtained through the kindness of Dr. Day. These tumors were examined macroscopically and microscopically, and cultures were made which were placed under the ordinary and anaërobic conditions. In all more than twenty specimens were examined. Swine and guinea-pigs have been inoculated with pure cultures of organisms isolated from the tumors, and one inoculation was made with a small piece of necrotic tissue from one of the growths.

The histology of the growths is comparatively uniform. As a rule, there are four distinct zones in a section cut perpendicularly to the surface, which correspond with the description of the gross specimen.

A section stained with hæmatoxylin and eosin shows externally a zone consisting of branching and spindle-shaped cells which stains well. The protoplasmic processes of the cells frequently anastomose. The intercellular substance stains feebly, taking, as a rule, an admixture of the acid and basic stains. Portions of the field may show a faintly striated eosin staining intercellular substance.

Next is a wide zone corresponding to the dense white tissue, which shows many spindle, oval, and roundish cells, the nuclei of which are granular and stain well. Karyokinetic figures are sometimes seen. With methylene-blue-orcein quite a few mastzellon are noted but no typical plasma cells. Bloodvessels are not numerous. Some are atypical, the walls being very thin. Others appear to be spaces in the tissue. There are many minute vessels in some of the sections having walls the cells of which stain deeply. The lumen of these vessels is not always uniform in size. Their general direction is away from the necrotic centre. They seem to be lymph channels. The softened contents of the cavities are necrotic and have no structure. A few faintly-stained nuclei and occasional normal nucleated cells are seen scattered throughout a granular eosin-staining field. There may be blue areas which, with the high power, are shown to be aggregations of intensely staining bodies of irregular shapes and various sizes, presumably nuclear fragments.

Between the necrotic and fibrous zones there is a narrow zone staining intensely with the basic stain. This also consists of nuclear fragments and homogeneous blue-staining material. The fibrous tissue contiguous to this necrobiotic zone is embryonal in character, in it there may be small areas of degeneration and necrosis. As a rule, the change from living to dead tissue is rather abrupt, as described above, but the transition may be much less clearly marked, and throughout the entire necrotic zone there may be nuclei which take the stain. In sections stained by Van Gieson's method for demonstrating connective tissue, the necrotic zone may give a picture resembling somewhat the arrangement of the fibres in the living tissue. Sections stained by Weigert's stain show elastic fibres in the more mature fibrous tissue.

In sections stained with methylene-blue or Gram-Weigert method the necrotic border of most of the cavities is seen to contain various bacteria, sometimes in enormous numbers. There may be bacilli of varying lengths as well as branching threads and cocci, singly, in pairs, groups, or chains. In some of the growths all these forms are present, in others there are very few organisms. Usually the living tissue is free from bacteria, except in the margins of the necrotic zone, the organisms being most numerous in the necrotic layer upon the walls of the cavities.

A small slender bacillus or diplococcus is the most frequent organism observed. Filaments or threads are seen in sections from most of the growths. These threads either stain uniformly or appear as strings of deeply staining spores or cocci in a less deeply staining field. Cultures were taken from the necrotic material of a number of the growths with varying results.

Usually from two to seven different organisms were isolated from each tumor. None of the organisms isolated were virulent enough to kill guinea-pigs. In a series of seven tumors a Gram-staining non-liquefying coccus was isolated twice, likewise a Gram-staining coccus which reduces glucose, twice. A ray fungus was isolated from three different tumors of the series of seven. Smears from all the growths show threads, some of them branching. Anaërobic cultures gave no uniform results, all of the organisms isolated being facultative.

From the morphological stand-point the ray fungus is the most interesting organism isolated. It grows on all the ordinary media and on vegetable infusions. It grows best in the incubator, but will grow at room temperature. Each of the ray fungi isolated shows certain minor differences, such as the rapidity of growth on various media, and in the growth on potato, some of them failing to grow on potato. All liquefy gelatin, and all stain by Gram's method. Swine were inoculated subcutaneously with pure cultures of the ray fungus, but the results were negative.

On October 26, 1899, a sixty-pound boar pig was inoculated by removing the right testicle, placing in the sac a small piece of necrotic material from tumor No. 5 of the series and sewing up the wound. The pig died November 20th, and a necropsy made the following day, as follows:

There are no changes in the solid organs and other viscera except a moderate amount of hypostatic congestion of the lungs, and some passive hyperæmia of the liver and spleen. The apparent anæmia of the organs is a striking feature. At the site of inoculation is a large indurated mass which is removed. As excised the dimensions are 20 x 16 x 10 cm. The mass on section is seen to be made up of a central area of softening and discoloration, and a surrounding capsule of dense scar tissue. The remaining testicle is partly within the area of necrosis, partly in the fibrous capsule, and a small portion external to the growth appearing normal. Histologically the changes already described are noted, viz., a central zone of necrosis and a zone of fibrous tissue. By the plate method a number of different organisms were isolated from this growth, but there were no colonies of the ray fungus.

The query naturally arises, Is not the growth an ordinary abscess due to a mixed infection? A definite answer cannot be given to the question at this stage of the work, but certain characteristics seem to point to the specific nature of it, viz.:

1. They never heal spontaneously.

2. Thorough drainage, such as is secured by a longitudinal incision carried deep enough to cut the tumor in two, does not result in a cure.

3. The peculiar manner in which the necrosis spreads regardless of lines of least resistance, as shown in the unremoved testicle of the pig inoculated with a portion of one of the growths, experimentally.

---

The Committee of Arrangements of the New Jersey Veterinary Medical Association for the Atlantic City meeting, September, 1901, held an important meeting in Jersey City, February 18, 1901. Dr. W. Herbert Lowe presided.

A tuberculin conference at Washington was recently held looking to a greater freedom of transportation of animals from Canada to the United States and Great Britain, relative to the tuberculin test.

